# Nomogram to predict rectal toxicity following prostate cancer radiotherapy

**DOI:** 10.1371/journal.pone.0179845

**Published:** 2017-06-22

**Authors:** Jean-Bernard Delobel, Khemara Gnep, Juan David Ospina, Véronique Beckendorf, Ciprian Chira, Jian Zhu, Alberto Bossi, Taha Messai, Oscar Acosta, Joël Castelli, Renaud de Crevoisier

**Affiliations:** 1 Dept. of Gastroenterology, Centre Hospitalier Universitaire Pontchaillou, Rennes, France; 2 LTSI, Inserm U1099, Université de Rennes 1, Rennes, France; 3 Centre Eugene Marquis, Rennes, France; 4 Escuela de Estadística, Universidad Nacional de Colombia, Medellin, Colombia; 5 Centre Alexis Vautrin, Vandoeuvre les Nancy, France; 6 Institut Gustave-Roussy, Villejuif, France; 7 Laboratory of Image Science and Technology, Southeast University, Nanjing, PR China; 8 Centre de Recherche en Information Biomédicale Sino-Français (CRIBs), Rennes, France; Taipei Medical University, TAIWAN

## Abstract

**Background:**

To identify predictors of acute and late rectal toxicity following prostate cancer radiotherapy (RT), while integrating the potential impact of RT technique, dose escalation, and moderate hypofractionation, thus enabling us to generate a nomogram for individual prediction.

**Methods:**

In total, 972 patients underwent RT for localized prostate cancer, to a total dose of 70 Gy or 80 Gy, using two different fractionations (2 Gy or 2.5 Gy/day), by means of several RT techniques (3D conformal RT [3DCRT], intensity-modulated RT [IMRT], or image-guided RT [IGRT]). Multivariate analyses were performed to identify predictors of acute and late rectal toxicity. A nomogram was generated based on the logistic regression model used to predict the 3-year rectal toxicity risk, with its accuracy assessed by dividing the cohort into training and validation subgroups.

**Results:**

Mean follow-up for the entire cohort was 62 months, ranging from 6 to 235. The rate of acute Grade ≥2 rectal toxicity was 22.2%, decreasing when combining IMRT and IGRT, compared to 3DCRT (RR = 0.4, 95%CI: 0.3–0.6, p<0.01). The 5-year Grade ≥2 risks for rectal bleeding, urgency/tenesmus, diarrhea, and fecal incontinence were 9.9%, 4.5%, 2.8%, and 0.4%, respectively. The 3-year Grade ≥2 risk for overall rectal toxicity increased with total dose (p<0.01, RR = 1.1, 95%CI: 1.0–1.1) and dose per fraction (2Gy *vs*. 2.5Gy) (p = 0.03, RR = 3.3, 95%CI: 1.1–10.0), and decreased when combining IMRT and IGRT (RR = 0.50, 95% CI: 0.3–0.8, p<0.01). Based on these three parameters, a nomogram was generated.

**Conclusions:**

Dose escalation and moderate hypofractionation increase late rectal toxicity. IMRT combined with IGRT markedly decreases acute and late rectal toxicity. Performing combined IMRT and IGRT can thus be envisaged for dose escalation and moderate hypofractionation. Our nomogram predicts the 3-year rectal toxicity risk by integrating total dose, fraction dose, and RT technique.

## Background

Rectal toxicity is among the main limiting side-effects of prostate cancer radiotherapy (RT), with negative impact on patient quality of life [1]. The incidence and severity of rectal toxicity after prostate cancer RT vary considerably among studies, primarily depending on patient and radiation characteristics, such as radiation dose and irradiated rectal volume.

The clear correlation between dose and tumor control in prostate cancer has historically justified a dose-escalation strategy (> 76Gy), which inevitably leads to increased doses delivered to normal surrounding tissues [2–4]. More recently, moderate hypofractionated regimens have been tested in localized prostate cancer [5–12]. Besides potentially increasing RT efficacy, the smaller number of hypofractionation treatments required enhances convenience for the patient, while decreasing health care costs. However, the increased fraction dose delivered to organs at risk (OAR) likely exposes patients to higher toxicity risks, the estimated α/β of the rectum for late toxicity ranging from 3 to 5Gy in the literature [13–15].

To reduce the doses to the OAR, irradiation techniques, such as intensity modulated radiation therapy (IMRT) and image guided radiation therapy (IGRT), have recently been combined. IMRT enables a highly conformal dose to be delivered to the concave shape of the prostate target, while limiting the dose to the convex shape of the rectum [16]. For over a decade now, IMRT has been shown to enable dose escalation to the prostate, without increasing genito-urinary (GU) or gastro-intestinal (GI) toxicity [17,18]. However, the prostate is significantly mobile, able to move (up to 2cm in the pelvis between fractions, thereby increasing the risk of prostate under-dosage and rectum over-dosage. This will enhance the risks of both tumor recurrence and rectal toxicity [19–21]. Using either intra-prostatic fiducial markers or onboard CT imaging (CBCT), IGRT seeks to correct prostate displacements prior to each treatment fraction.

Given this context, quantifying the relative impact of the combined parameters like total dose, fraction dose, and radiotherapy technique (IMRT, IGRT) on the risk of both acute and late rectal toxicity appears justified, particularly with the aim to generate a reliable predictive tool for rectal toxicity. This tool can be used for both informing the patient, as well as guiding the physician in decision-making process on prostate cancer treatment options.

We thus conducted this study involving a large-scale patient population undergoing RT for prostate cancer, pursuing the following objectives:

- to quantify the incidence and severity of acute and late rectal toxicity;
- to identify predictors of rectal toxicity, incorporating both patient and treatment characteristics, such as recent RT techniques (IMRT and IGRT), dose escalation (up to 80 Gy), and moderate hypofractionated RT (2.5 Gy/fr);
- to create a nomogram able to assess the risk of rectal toxicity for a given patient in routine practice.

## Methods

### Patient and tumor characteristics

A total of 972 patients undergoing definitive radiotherapy for localized prostate adenocarcinoma were included in this study. Data were prospectively compiled for 487 patients (50%) treated in 17 French institutions between 2000 and 2012 in two randomized trials, namely GETUG 06 comparing 70Gy with 80Gy [22] and STIC-IGRT testing two IGRT frequencies [23], and retrospectively recorded for 485 other patients treated in two institutions in order to assess only the fractionation impact. All patients exhibited a prostate adenocarcinoma proven by biopsy. Pretreatment evaluations consisted of clinical history, physical examination, laboratory studies, CT scan, and bone scan. Patients were classified into prognostic risk groups according to pretreatment PSA levels, clinical stage (T1–4), and Gleason score, as described by D’Amico [24]. Patient and tumor characteristics are provided in Table 1.

**Table 1:** Patient, tumor, and treatment characteristics.

**Patient characteristics**				
		Prospective cohort	Retrospective cohort	Total cohort
Number of patients		487	485	972
Age (year), mean (range)		68 (48–83)	70 (45–83)	69 (45–83)
Diabetes mellitus, %		9	9	9
Anticoagulant treatment, %		28	22	24
Hypertension, %		24	18	21
Coronary insufficiency, %		3.5	14	9
Prior abdominal or pelvic surgery, %		28	37	34
**Tumor characteristics**				
PSA (ng/ml), mean (range)		13 (0.3–84)	13 (0.5–79)	13 (0.3–84)
T stage, %	T1	22	45	33
	T2	65	48	57
	T3	13	7	10
Prognostic risk group, %	Low	12	20	16
	Intermediate	62	55	58
	High	26	25	26
**Treatment characteristics**				
***Radiotherapy technique***				
Standard 3D conformational RT, %		68	92	80
IMRT alone, %		4	8	7
IMRT combined with IGRT, %		28	0	13
***Total dose and fractionation***				
70Gy, %	2.5Gy/fr, 4 fr/week	0	58	29
2Gy/fr, 5 fr/week	34	24	29
78-80Gy, %	2Gy/fr, 5 fr/week	66	18	42
***Target volume***				
Prostate only, %		12	20	16
Prostate + seminal vesicles, %		88	80	84
***Rectum dosimetric parameters***				
Dmax (Gy), mean (range)		74 (62–79)	74 (69–79)	74 (62–79)
D25 (Gy) mean (range)		60 (36–77)	65 (31–74)	61 (31–77)
D50 (Gy) mean (range)		41 (15–78)	47 (21–66)	44 (15–78)
***Androgen deprivation therapy*** (concomitant and adjuvant) %		16.4	6	11

Gy, Gray; fr, fraction; IMRT, intensity modulated radiotherapy; IGRT, image-guided radiotherapy; PSA, prostate specific antigen.

Retrospective data were obtained from medical records in a fully anonymized and de-identified manner. The authors had no access to identifying information. Prospective data (from GETUG 06 and STIC-IGRT studies) were fully anonymized, and all patients provided informed consent. The STIC-IGRT protocol was approved by the Kremlin Bicêtre Hospital ethics committee (CPP) on February 2, 2007 (Project n°07–002), and the GETUG 06 protocol by the Lorraine ethics committee CCPPRB, on June 22, 1999.

### Radiotherapy description

Details on treatment techniques are presented in Table 1.

All patients received 3D conformational RT (3DCRT), carried out in accordance with the French GETUG group recommendations [22]. Each patient underwent simulation and treatment in supine position. Target volume and organs at risk, namely the bladder, rectum, and femoral heads, were manually delineated on 3mm- to 5mm-thickness CT slices. The planning target volume (PTV) was calculated as including the prostate ± seminal vesicles, with a 10mm additional margin in each space direction, except posteriorly where it was reduced to 5mm in order to spare the rectum. The PTV margins were the same for 3DCRT and IMRT alone, and combined IMRT plus IGRT treatments, according to the GETUG recommendations [22]. The seminal vesicles were not irradiated in low-risk tumors. The pelvic lymph nodes were not treated. The rectum was manually delineated from 2cm above to 2cm below the prostate and seminal vesicles. The rectal wall was generated with a 5mm thickness from the external manually-delineated rectal contour.

The total dose delivered to the prostate was either 70Gy (58%) or 78 to 80Gy (42%). The total dose received by the seminal vesicles was 46Gy in all cases. The dose per fraction was either 2Gy per fraction, five fractions per week (71%), or 2.5Gy per fraction, four fractions per week (29%). In order assess the impact of the moderate hypofractionated scheme, a series of 401 patients treated in a single institution was included, all having received a total dose of 70 Gy over 7 weeks with 3DCRT, either at 2Gy per fraction with five fractions per week or at 2.5Gy per fraction with four fractions per week. Dose parameters between the treatment techniques and their comparison are displayed in Table 2.

**Table 2:** Dose parameters between the radiotherapy techniques.

Organ	Dose parameters	3DCRT	IMRT	P value*
Prostate	Prescribed total dose (Gy), mean (range)	75 (70–80)	79 (70–80)	<0.01
Rectum	Dmax (Gy), mean (range)	74 (69–81)	75 (62–81)	<0.01
	D25 (Gy), mean (range)	64 (31–77)	57 (36–72)	<0.01
	D50 (Gy), mean (range)	46 (15–78)	39 (24–66)	<0.01

Gy: Gray; 3DCRT: 3D conformal radiotherapy; IMRT: intensity modulated radiotherapy

*P values were calculated using the Mann Whitney test comparing the dose parameters of the treatment techniques

IMRT and IGRT (fiducial markers or CBCT) were applied to 20% and 13% of all patients, respectively. Fiducial markers in IGRT were always used in combination with IMRT and applied to patients having received 78-80Gy to the prostate. Three fiducial markers were implanted in the prostate. Two orthogonal kV images were acquired. The fiducial markers were then registered between kV images and digital reconstructed radiographs. In the absence of fiducials, a prostate registration was performed between CBCT and the planning CT. In the subgroup of patient treated with 78-80Gy, the choice of RT technique used could be classified into three increasing complexity levels: standard 3DCRT for 54%, IMRT only for 15%, and IMRT combined with IGRT for 31%.

### Follow-up and toxicity grading

Patients were assessed weekly during treatment, then every 3 months over 1 year, and every 6 months thereafter. To determine the severity and incidence of principal rectal complaints, patient data were compiled either prospectively based on standardized questionnaires or retrospectively from physicians’ medical files, filled in at every follow-up visit. Acute toxicity was defined as adverse events occurring either during treatment or within 3 months of treatment completion, recorded according to RTOG [25] and CTCAE V3.0 [26] toxicity grading. Rectal toxicity was defined as adverse events occurring within the 3 months after treatment completion. Rectal complaints were classified according to different symptoms, such as rectal bleeding, proctitis (urgency, tenesmus), diarrhea, and fecal incontinence, in compliance with the LENT-SOMA morbidity scoring system [27].

### Statistical analysis

The Kaplan-Meier method was applied to calculate the cumulative risk of Grade ≥2 rectal toxicity. The impact of the following parameters on acute and late rectal toxicity was assessed: patient-related parameters (age, diabetes mellitus, anticoagulant treatment, arterial hypertension, coronary insufficiency, and prior abdominal surgery), tumor-related parameters (T-stage and risk group), and radiation-related parameters (total dose, dose per fraction, RT technique [3DCRT, IMRT, and IGRT], and rectal dose), as well as androgen deprivation. Regression logistic was used for univariate and multivariate analyses. Covariates included in the multivariate model were those with a p value <0.2 (significant or trend p values) in univariate analysis. Displayed variables in the multivariate analyses were those with p ≤0.05. The analysis was performed on the prospective, the retrospective and the whole cohorts. Differences between survival curves were assessed using the log-rank test. Besides, the Mann-Whitney test was applied to compare the dosimetric values depending on the treatment technique (Table 2). The statistical significance level was 0.05.

A nomogram was drawn based on the logistic regression model in order to predict the 3-year rectal toxicity (Grade ≥2) risk, with its accuracy verified by dividing the cohort into training (70% of patients) and validation (30% of patients) subgroups. The logistic regression model parameters were estimated using the training group, then applied to predict the complication probability of patients in the validation subgroup. Concordance C-index and actual *versus* predicted probability fit were employedto assess the nomogram accuracy.

The analyses were performed using the SPSS V18 (Chicago, IL) and R, by means of the rms package.

## Results

Mean follow-up for the entire cohort was 62 months, ranging from 6 to 235. The mean follow-ups according to the fraction dose and the RT technique employed are displayed in Tables 3 and 4, respectively.

**Table 3:** Follow-up and 3-year rectal toxicity (Grade ≥2) rate according to the fraction dose when delivering 70 Gy to the prostate with 3DCRT.

Fraction dose (Gy)		2 (n = 277)	2.5 (n = 283)
**Follow-up (in months, mean and range values)**		70 (6–155)	59 (6–235)
**Overall rectal toxicity (Grade≥2)**	Rate (%)*	9.5 (5.9–13.1)	13.7 (9.5–17.9)
	P value and RR (95%CI)*	p = 0.03; RR = 3.3 (95%CI: 1.1–10.0)	

*The 3-year toxicity rates (with 95%CI) were calculated using the Kaplan-Meier method. The binary logistic regression test was used to assess the impact of treatment parameters on rectal toxicity risk. The relative risks (RR) with 95% confidence interval (CI) and P value are given.

**Table 4:** Follow-up and 3-year rectal toxicity (Grade ≥2) rate according to the treatment technique employed when delivering high dose (78–80 Gy) at a standard fractionation (2 Gy).

Technique		3DCRT (n = 220)	IMRT alone (n = 63)	IMRT and IGRT (n = 128)
**Follow-up (in months, mean and range values)**		76 (10–152)	54 (12–108)	31 (6–74)
**Overall rectal toxicity (grade ≥2)**	Rate (%)*	19.4 (14.0–24.8)	13.1 (4.5–21.7)	4.1 (0–8.3)
	P value and RR (95%CI)*	p = 0.01; RR = 0.5 (95%CI: 0.3–0.8)		

*The 3-year toxicity rates (with 95%CI) were calculated using the Kaplan-Meier method. The binary logistic regression test was used to assess the impact of treatment parameters on rectal toxicity risk. The relative risks (RR) with 95% confidence interval (CI) and P value are given.

### Quantification of acute rectal toxicity

Overall, 35.9% of patients exhibited only Grade 1 rectal toxicity during radiotherapy, while maximum acute rectal toxicity was recorded as Grades 2 and 3 in 20.7% and 1.5% of patients, respectively. The rate of acute Grade ≥2 rectal toxicity was therefore 22.2%. The primary acute toxicity events were diarrhea and urgency/tenesmus, with Grade ≥2 events affecting 8.3% and 5.5% of patients, respectively. No patient experienced Grade ≥2 acute rectal bleeding or fecal incontinence.

### Quantification of late rectal toxicity

**Fig 1:**
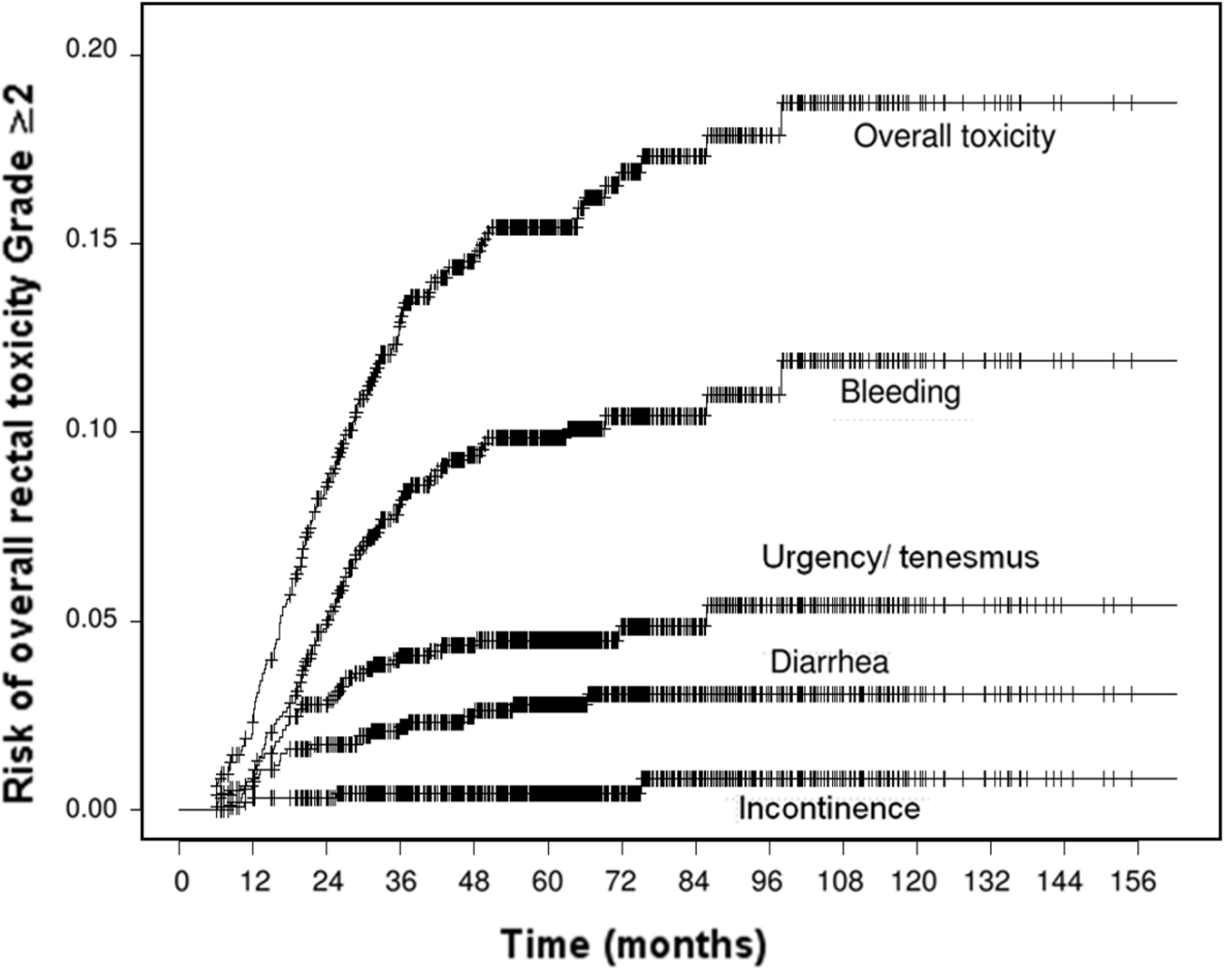
Risk of late rectal toxicity (Grade ≥2), overall and by symptoms (SOMA-LENT classification).

Fig 1 displays the cumulative risks of Grade ≥2 rectal toxicity, both overall and classified by symptom. At 5-year follow-up, these rates were 15.3% (95%CI: 12.9–17.7) for overall toxicity, 9.9% (95%CI: 7.9–11.9) for rectal bleeding, 4.5% (95%CI: 3.4–5.9) for urgency/tenesmus, 2.8% (95%CI: 1.6–4.0) for diarrhea and 0.4% (95% CI: 0–1.0) for fecal incontinence, respectively.

The 5-year Grade 3 overall rectal toxicity rate was 3.5% (95% CI: 2.3–4.7), no patient exhibiting Grade 4 toxicity.

The following data are based on multivariate analysis and concern Grade ≥2 toxicity, with significant parameters highlighted.

### Factors impacting the risk of acute rectal toxicity (Grade ≥2)

The risk of Grade ≥2 acute rectal toxicity was reduced when combining IMRT and IGRT (3DCRT *vs*. IMRT alone *vs*. IMRT+IGRT) (p<0.01). Compared to 3DCRT, acute rectal toxicity was decreased when combining IMRT and IGRT (RR = 0.43, 95%CI: 0.29–0.64, p<0.01). Results of univariate and multivariate logistic regression are given in Table 5.

**Table 5:** Parameters impacting on the risk of acute rectal toxicity (Grade ≥2) in the prospective cohort (n = 487).

		Acute rectal toxicity	
	Covariates	Univariate analysis (P value)	Multivariate analysis (P value)
**Patient characteristics**	Age	0.711	-
	Diabetes mellitus	0.998	-
	Anticoagulant	0.958	-
	Hypertension	0.264	-
	Coronary insufficiency	0.817	-
	History of abdominal surgery	0.480	-
**Tumor characteristics**	T-stage	0.220	-
	Risk group	0.619	-
**Treatment characteristics**	Total dose	0.047	-
	RT techniques*	<0.001	<0.001
	Androgen deprivation therapy	0.048	-

P values were calculated using the univariate and multivariate logistic regressions.

*Three radiotherapy techniques were tested: 3DCRT, IMRT alone, and IMRT combined with IGRT.

When delivering high doses to the prostate (78–80 Gy), the percentages of patients presenting toxicity were 7% when applying both IGRT and IMRT, 14% for IMRT alone, and 28% for 3DCRT.

### Factors impacting the risk of late rectal toxicity (Grade ≥2) and corresponding nomogram

**Fig 2:**
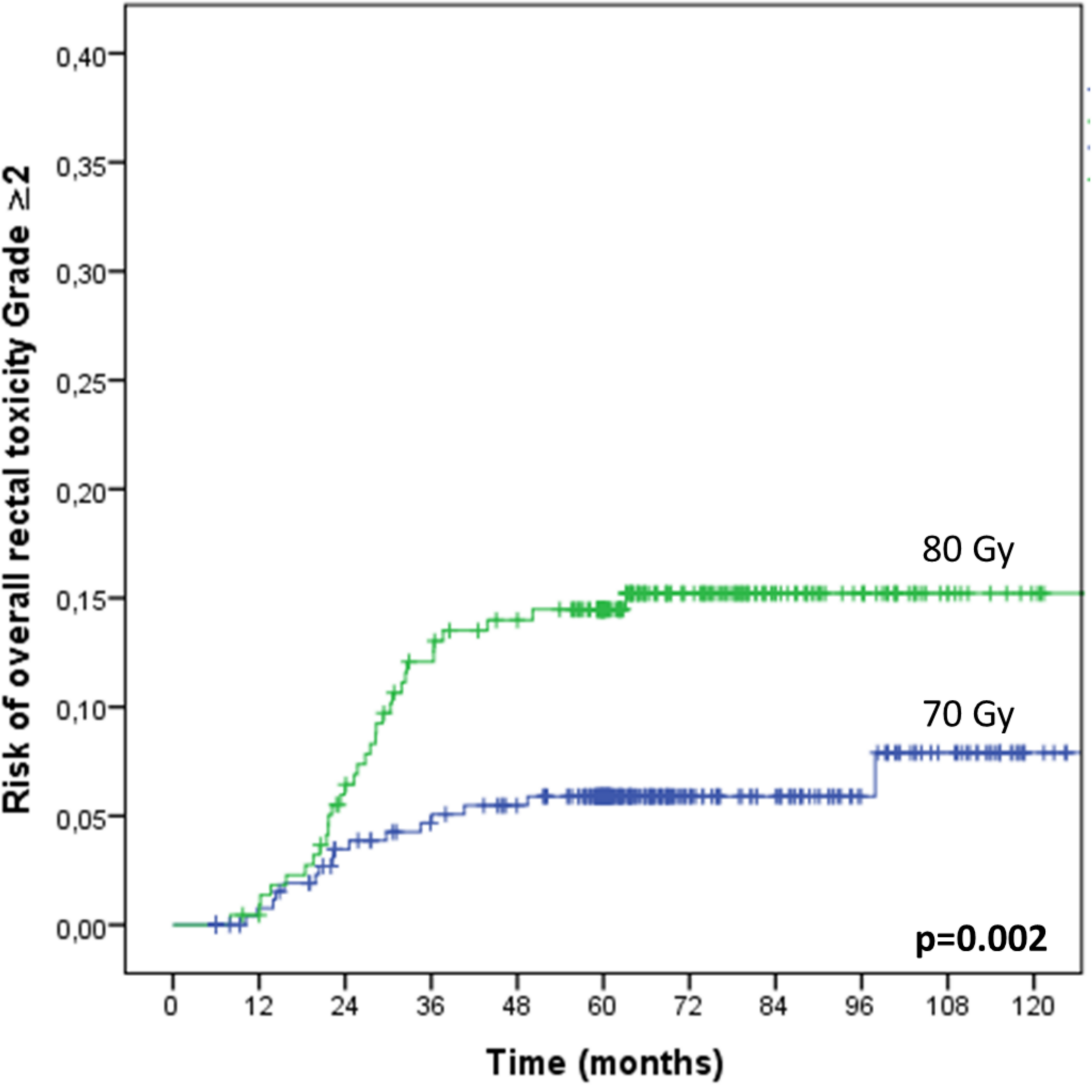
Impact of dose escalation on the Grade ≥2 overall rectal toxicity risk. The patients (n = 491) received either a total dose of 70Gy (n = 277) or 78-80Gy (n = 220), using the same 3D conformal RT technique.

**Fig 3:**
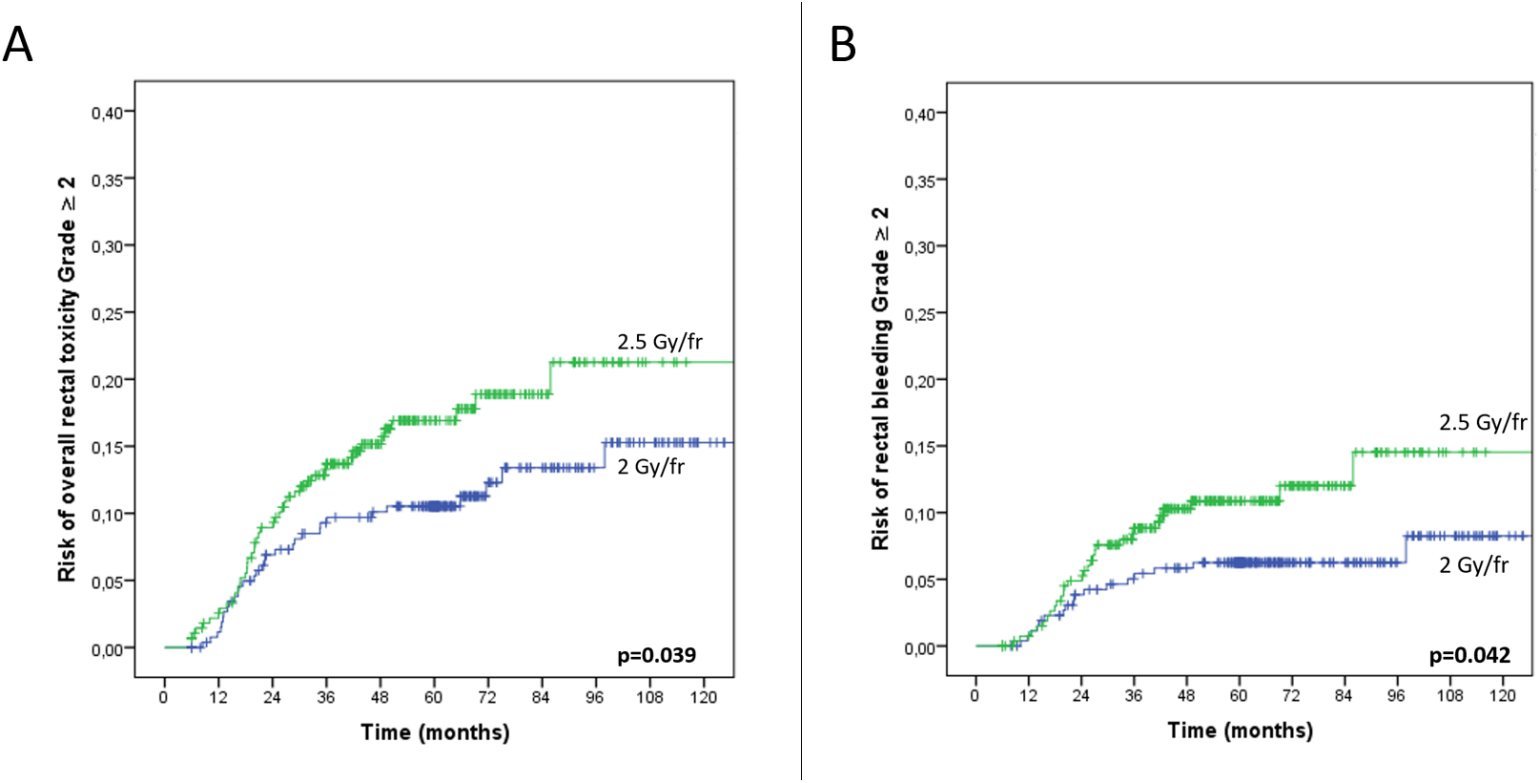
Impact of hypofractionation on the Grade ≥2 overall rectal toxicity (Fig 3A) and rectal bleeding risks (Fig 3B). The patients (n = 555) received a total dose of 70 Gy with the same 3D conformal RT technique, at either 2 Gy/fr (n = 272) or 2.5 Gy/fr (n = 283).

**Fig 4:**
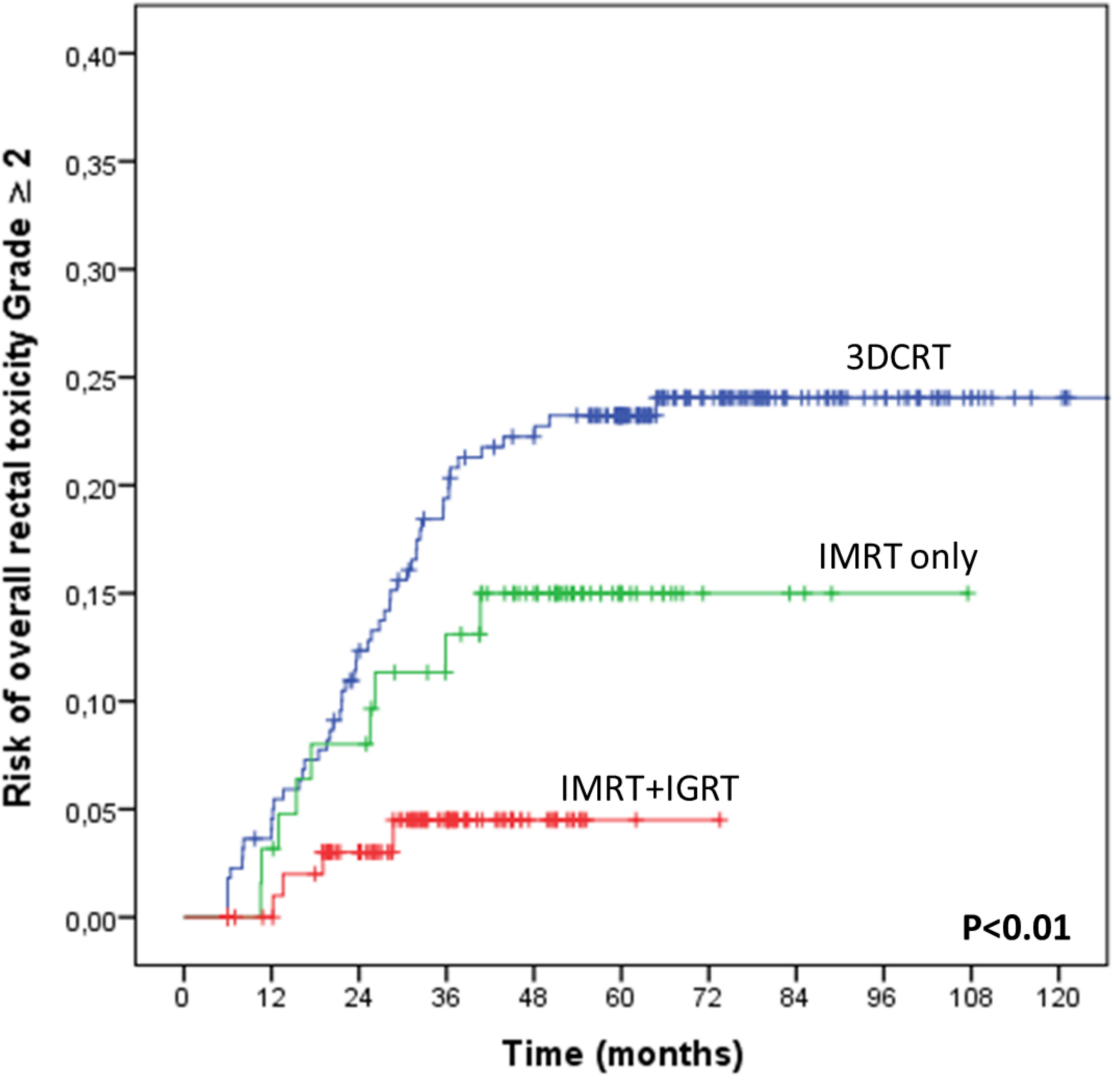
Impact of the radiation technique on the overall rectal toxicity (Grade ≥2) risk when delivering high doses to the prostate. The patients (n = 401) received a total dose of 78–80 Gy, by means of either 3DCRT (n = 220) or IMRT alone (n = 63), or by combining IMRT and IGRT (n = 128).

The 3-year overall rectal toxicity risk increased with the total dose (P = 0.001, RR = 1.09, 95%CI: 1.03–1.15) and dose per fraction (2Gy *vs*. 2.5Gy) (P = 0.03, RR = 3.29, 95%CI: 1.09–9.98), while decreasing when combining IMRT and IGRT (3DCRT *vs*. IMRT alone *vs*. IMRT+IGRT) (P = 0.007, RR = 0.47, 95%CI: 0.27–0.81). Compared to 3DCRT, the 3-year rectal toxicity risk decreased when combining IMRT and IGRT (P = 0.013, RR = 0.40, 95% CI: 0.19–0.83). The impacts of the total dose, fraction dose, and RT technique on the overall rectal toxicity and on the rectal bleeding risks are displayed in Figs 2, 3A, 3B and 4, respectively. The 3-year rectal toxicity rates according to fraction dose and technique are displayed in Tables 3 and 4, respectively. Parameter results upon univariate and multivariate logistic regressions for 3-year overall rectal toxicity (Grade ≥2) risk are given in Table 6.

**Table 6:** Parameters impacting on the 3-year risk of late rectal toxicity (Grade ≥2).

		Retrospective cohort (n = 485)		Prospective cohort (n = 487)		Whole cohort (n = 972)	
	Parameters	Univariate analysis (P value)	Multivariate analysis (P value)	Univariate analysis (P value)	Multivariate analysis P value)	Univariate analysis (P value)	Multivariate analysis (P value)
**Patient characteristics**	Age	0.873	-	0.099	-	0.32	-
	Diabetes mellitus	0.752	-	0.652	-	0.96	-
	Anticoagulant	0.964	-	0.754	-	0.86	-
	HTA	0.302	-	0.382	-	0.12	-
	Coronary insufficiency	0.966	-	0.623	-	0.72	-
	History of abdominal surgery	0.667	-	0.982	-	0.69	-
**Tumor characteristics**	T-stage	0.167	-	0.43	-	0.28	-
	Risk group	0.817	-	0.042	-	0.23	-
**Treatment characteristics**	Total dose	0.589	-	0.133	0.034	0.15	0.001
	Dose per fraction	0.01	0.004	NA	-	0.04	0.03
	RT techniques*	NA	-	0.024	0.01	0.038	0.007
	Androgen deprivation therapy	0.745	-	0.65	-	0.47	-

HTA, arterial hypertension; NA, non applicable (all the patients of the prospective cohort were treated at 2 Gy/fraction, and IGRT was not used in the retrospective cohort). P values were calculated using the regression logistic analysis for each symptom.

*Three radiotherapy techniques were tested: 3DCRT, IMRT alone, and IMRT combined with IGRT.

**Fig 5:**
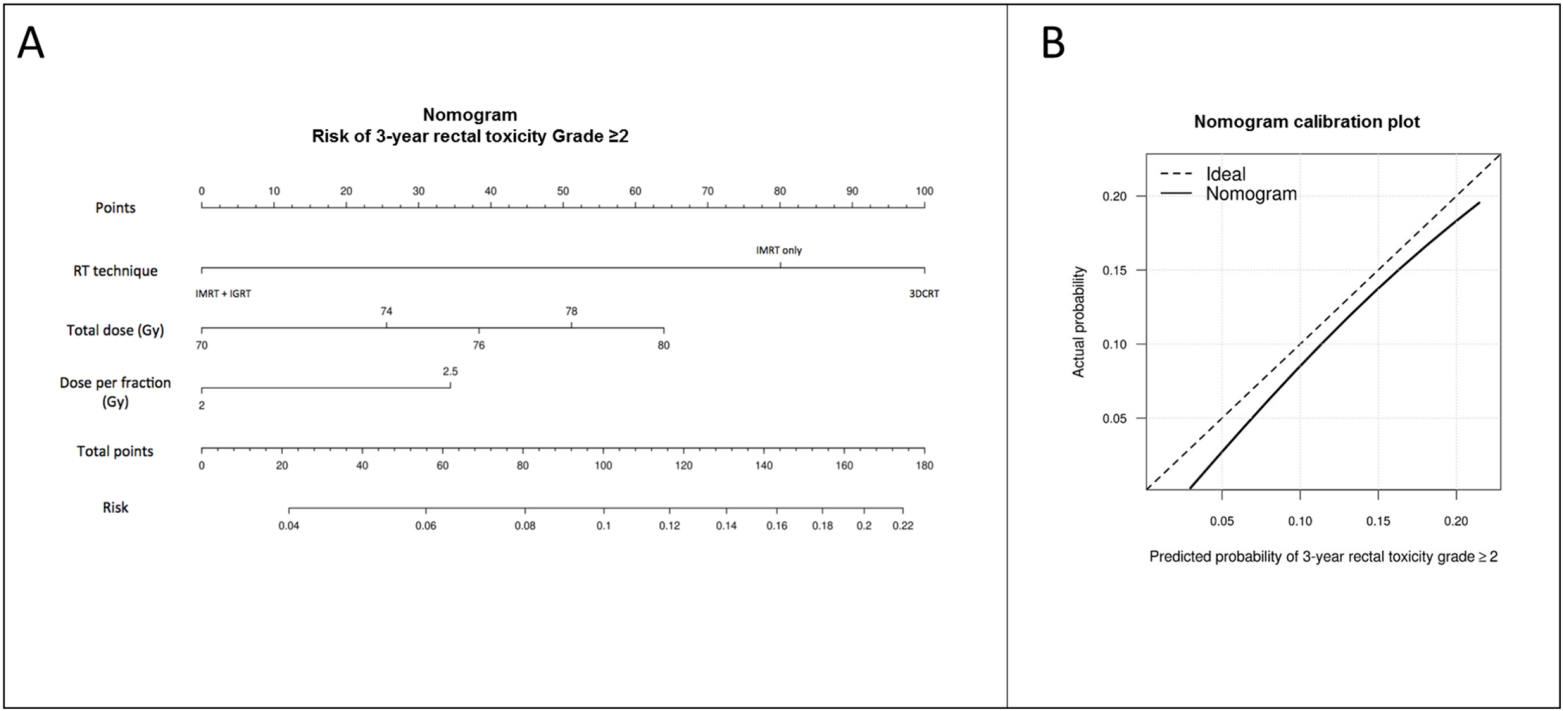
Nomogram and calibration plot (validation cohort) for the 3-year Grade ≥2 overall late rectal toxicity risk. 3DCRT: 3D conformal radiotherapy, IGRT: image-guided radiotherapy, IMRT: intensity-modulated radiotherapy. To use the nomogram, align a straight edge so that it intersects with each predictor line (RT technique, total dose, or dose per fraction), then read the corresponding "Points" on the first line for each predictor. Add the points of the three predictors in order to calculate the total points. Align your straight edge to the "Total points" line and read the toxicity "Risk" on the last line. For example, a patient treated with IMRT and IGRT (0 points) to a total dose of 80 Gy (65 points) at 2 Gy (0 points) per fraction has a risk of late rectal toxicity of 7% (65 points). Calibration plot to assess the nomogram performance by a nonparametric fit of the predicted probability versus actual observed probability in the validation cohort. The corresponding C-index is 60%.

The corresponding nomogram and calibration plot in the validation cohort are illustrated in Fig 5. The C-index was 60%.

Based on the prospective cohort only, the 3-year overall rectal toxicity risk significantly increased with the total dose (p = 0.034, RR = 1.1, 95%CI: 1.0–1.1), and decreased when combining IMRT and IGRT (p = 0.01, RR = 0.5, 95% CI: 0.2–0.8).

## Discussion

Our study analyzed the risk of rectal toxicity based on a large cohort of patients undergoing radiotherapy for prostate cancer. The most common symptoms of acute and late rectal toxicity were rectal bleeding, proctitis (urgency/tenesmus) and diarrhea respectively, in line with previous publications [22,28]. Our findings confirmed the dose-effect relationship as regards rectal toxicity, demonstrating that combining IMRT and IGRT dramatically decreased the risks of both acute and late rectal toxicity. Even after increasing the total dose up to 80Gy to be delivered to the prostate, the risks of Grade ≥2 acute or late rectal toxicity were <10% when combining the two techniques. These risks were not superior to those calculated when delivering 70Gy by means of 3DCRT. Our nomogram (Fig 5) thus permitted the individual prediction of overall rectal toxicity risk, based on these parameters, namely total dose, fraction dose, and RT technique.

Unlike the urinary toxicity risk, which was shown to increase linearly following treatment, the risk of rectal toxicity plateaued at the 3-year mark in our study (Fig 1), in line with a previously published report [29]. Our data suggest that the patient can possibly be reassured that, once this time point has been reached, he is most unlikely to develop new radiation rectal toxicity events. Rectal bleeding was the principal side-effect observed, while diarrhea and urgency/tenesmus were much less common, and fecal incontinence a rare occurrence. Given that pelvic lymph nodes were not included in the target volume, the small bowel was not irradiated, potentially accounting for the low incidence of diarrhea. The observed GI toxicity was therefore mainly associated with the dose received by the rectum and anal canal. Other studies, however, observed slightly higher rates of fecal incontinence requiring pads, with 3-year rates reported at around 3 to 9% [29,15,30].

Increasing total dose to be delivered to the prostate from 8 to 10Gy significantly increased the risk of overall rectal toxicity by around 10% in our series, as was the case in most randomized studies [2–4], and in a meta-analysis involving 2,812 patients [31].

The pathophysiology of "overall" rectal toxicity varies clearly with each symptom. Since bleeding is caused by telangiectasias, functional symptoms like urgency or tenesmus are due to changes in rectal compliance, and incontinence to loss of sphincter function. From a radiobiological point of view, most dosimetric analyses indicate a prevalently serial-like behavior of rectal bleeding incidences [15], which demonstrates that bleeding is primarily accounted for by the highest dose delivered to the anterior rectal wall rather than by the "average" dose delivered to the full rectum. This fact is also strongly supported by endoscopic telangiectasia findings, revealing an increase in telangiectasias from the posterior to the anterior rectum wall, with Grade 3 telangiectasias mostly limited to the high dose region of the anterior rectum wall [32]. More recently, the inferior–anterior ano-rectum region has been identified as highly predictive of rectal bleeding, by means of an elastic registration method [33,34]. These findings suggest that attempts should be made to minimize the dose delivered to the anal canal and inferior rectum, whilst not only restricting the highest dose delivered to the whole rectum.

Moderate hypofractionated regimens have clearly shown their non-inferiority in terms of biochemical control, as compared with standard fractionation. Overall, eight randomized studies assessed the benefits of moderate hypofractionation (2.4 to 3.4Gy/fr) administered in a reduced number of fraction (19 to 30 fractions) and treatment duration (4 to 6.5 weeks), while simultaneously decreasing the total dose (52.5Gy to 72Gy) [5–12]. The reference RT arm was delivered a total dose ranging from 64 to 80Gy at a standard fraction dose of either 1.8Gy/fr or 2Gy/fr. While the late rectal toxicity risks did not differ between the two arms in most studies [5,7,9,10,12,35], the HYPRO and RTOG 0415 trials revealed, however, a significantly increased risk of acute and late rectal toxicity in the hypofractionation schedule arm [6,36], without confirming the initially made radiobiological hypothesis.

The results obtained with our hypofractionated regimen can be compared to those of the RTOG 0415 trial that used the same fractionation, *i*.*e*., 2.5Gy/fr, and same total dose of 70Gy [6]. Both studies found a higher risk of Grade ≥2 late rectal toxicity with the hypofractionated regimen compared to the standard fractionation (RR = 3.3 in our study and 1.6 in the RTOG 0415 trial). Whilst using hypofractionation, the 5-year Grade ≥2 rectal toxicity rates were similar in both studies, namely 16.9% in our study and 22.4% in the RTOG study.

Our study revealed that combining both IMRT and IGRT greatly decreased the risk of both acute and late rectal toxicity, particularly when high doses were delivered to the prostate (Fig 4). Whereas the benefits of IMRT when decreasing the doses delivered to the rectum, thus GI toxicity, have been well documented [16,18], those of IGRT have been less well assessed in the literature. No randomized or prospective studies have been conducted so far to draw comparisons between 3DCRT or IMRT and IGRT. In line with our study data, five retrospective analyses [37–41] found a decrease in both acute GI and GU toxicity [41], as well as late GU [37,39,40] and GI [38–40] toxicity.

This study exhibits several limitations that we wish to emphasize. In order to assess the impact of moderate hypofractionation, we combined retrospective and prospective data, whereas only prospective patients underwent standard fractionation. Our retrospective data were, nevertheless, very informative, given that they originated from a single institution that irradiated all prostate cancers using both the same 3DCRT technique and total dose (70 Gy), within the same time period (7 weeks). Nonetheless, for practical reasons, fractionation was set at either 2Gy/fraction (five fractions/week), or 2.5Gy/fraction (four fractions/week). In spite of this, our full analysis was conducted in two stages, while either excluding (for the vast majority of data) or including this retrospective data (only to assess the impact of moderate hypofractionation). Besides, mean follow-up of the IGRT cohort was inferior than that of the others (31 months *vs*. 54 and 76 months), which did not allow us to generate nomograms exceeding 3 years of follow-up. Furthermore, we did not include planning parameters like dose-volume histograms (DVH) into the analyses, as our study was aimed to generate pre-planning nomograms, as a tool to guide oncologists in their decision-making process as to treatment options, *i*.*e*., performing radical prostatectomy or radiotherapy. Nevertheless, using patient DVH-based metrics would have strongly increased the individual predictability, as normal tissue complication probability (NTCP) models [15,42,43]. Lastly, our study did not investigate the availability of biological markers for rectal toxicity. In brief, fecal excretion of human DNA and calprotectin were shown to increase upon pelvic treatment and could thus be considered as biomarkers for intestinal toxicity [44]. On the other hand, while blood citrulline was found to decrease upon pelvic radiotherapy, variations in citrulline levels were not correlated to mucosal toxicity [45]. Genetic predisposition for late rectal bleeding had previously been reported, concerning single nucleotide polymorphisms [46–48] or reduced gene expression [49–51]. The combination of all these factors, namely genetic susceptibility, patient characteristics, RT technique, and dosimetric data, may have contributed to strongly increase the predictability of radio-induced rectal injury.

## Conclusions

This study revealed that acute and late rectal toxicity was significantly decreased when combining recent RT techniques like IMRT and IGRT. Such a combined IMRT-IGRT approach can be therefore considered as the standard RT technique when administering high dose (>76 Gy) or moderate hypofractionated radiation therapy for prostate cancer therapy. Yet these conclusions are not based on a randomized control trial. For this reason, the nomogram we generated requires further validation by means of external data. Having said this, we wish to emphasize that it proves to be a simple and helpful tool for decision-making and patient information.
